# Supplementary material for: HIVprotI: an integrated web based platform for prediction and design of HIV proteins inhibitors
Source: J Cheminform. 2018 Mar 9;10:12. doi: 10.1186/s13321-018-0266-y (PMC5845081; doi:10.1186/s13321-018-0266-y)
Supplement: Supplementary file 2 — Additional file 2. Source code of HIVProtI web server. [file 13321_2018_266_MOESM2_ESM.zip › HIVprotI_Source-code/hivprotI/weka-3-6-6/doc/weka/gui/explorer/PreprocessPanel.html]

PreprocessPanel


---


|  |  |  |
| --- | --- | --- |
| **Overview** \| **Package** \| **Class** \| **Tree** \| **Deprecated** \| **Index** \| **Help** \| **Weka's home** | |  |
| **PREV CLASS**   **NEXT CLASS** | **FRAMES**    **NO FRAMES**     **All Classes** |  |
| SUMMARY: NESTED \| FIELD \| CONSTR \| METHOD | DETAIL: FIELD \| CONSTR \| METHOD |  |


---


## weka.gui.explorer Class PreprocessPanel

```
java.lang.Object
  java.awt.Component
      java.awt.Container
          javax.swing.JComponent
              javax.swing.JPanel
                  weka.gui.explorer.PreprocessPanel
```

**All Implemented Interfaces:**: java.awt.image.ImageObserver, java.awt.MenuContainer, java.io.Serializable, java.util.EventListener, javax.accessibility.Accessible, Explorer.CapabilitiesFilterChangeListener, Explorer.ExplorerPanel, Explorer.LogHandler

---

``` public class PreprocessPanel extends javax.swing.JPanel implements Explorer.CapabilitiesFilterChangeListener, Explorer.ExplorerPanel, Explorer.LogHandler ```

This panel controls simple preprocessing of instances. Summary
information on instances and attributes is shown. Filters may be
configured to alter the set of instances. Altered instances may
also be saved.

**Version:**
:   $Revision: 7529 $

**Author:**
:   Richard Kirkby (rkirkby@cs.waikato.ac.nz), Len Trigg (trigg@cs.waikato.ac.nz)

**See Also:**: Serialized Form

---

**Nested Class Summary**

**Nested classes/interfaces inherited from class javax.swing.JComponent**

`javax.swing.JComponent.AccessibleJComponent`

**Nested classes/interfaces inherited from class java.awt.Component**

`java.awt.Component.BaselineResizeBehavior`


**Field Summary**

**Fields inherited from class javax.swing.JComponent**

`TOOL_TIP_TEXT_KEY, UNDEFINED_CONDITION, WHEN_ANCESTOR_OF_FOCUSED_COMPONENT, WHEN_FOCUSED, WHEN_IN_FOCUSED_WINDOW`

**Fields inherited from class java.awt.Component**

`BOTTOM_ALIGNMENT, CENTER_ALIGNMENT, LEFT_ALIGNMENT, RIGHT_ALIGNMENT, TOP_ALIGNMENT`

**Fields inherited from interface java.awt.image.ImageObserver**

`ABORT, ALLBITS, ERROR, FRAMEBITS, HEIGHT, PROPERTIES, SOMEBITS, WIDTH`


**Constructor Summary**

`PreprocessPanel()`
  
          Creates the instances panel with no initial instances.


| **Method Summary** | |
| --- | --- |
| `void` | `addPropertyChangeListener(java.beans.PropertyChangeListener l)`             Adds a PropertyChangeListener who will be notified of value changes. |
| `void` | `addUndoPoint()`             Backs up the current state of the dataset, so the changes can be undone. |
| `void` | `capabilitiesFilterChanged(Explorer.CapabilitiesFilterChangeEvent e)`             method gets called in case of a change event |
| `void` | `edit()`             edits the current instances object in the viewer |
| `void` | `generateInstances()`             sets Instances generated via DataGenerators (pops up a Dialog) |
| `Explorer` | `getExplorer()`             returns the parent Explorer frame |
| `Instances` | `getInstances()`             Gets the working set of instances. |
| `java.lang.String` | `getTabTitle()`             Returns the title for the tab in the Explorer |
| `java.lang.String` | `getTabTitleToolTip()`             Returns the tooltip for the tab in the Explorer |
| `static void` | `main(java.lang.String[] args)`             Tests out the instance-preprocessing panel from the command line. |
| `void` | `removePropertyChangeListener(java.beans.PropertyChangeListener l)`             Removes a PropertyChangeListener. |
| `void` | `saveInstancesToFile(AbstractFileSaver saver, Instances inst)`             saves the data with the specified saver |
| `void` | `saveWorkingInstancesToFileQ()`             Queries the user for a file to save instances as, then saves the instances in a background process. |
| `void` | `setExplorer(Explorer parent)`             Sets the Explorer to use as parent frame (used for sending notifications about changes in the data) |
| `void` | `setInstances(Instances inst)`             Tells the panel to use a new base set of instances. |
| `void` | `setInstancesFromDB(InstanceQuery iq)`             Loads instances from a database |
| `void` | `setInstancesFromDBQ(java.lang.String url, java.lang.String user, java.lang.String pw, java.lang.String query)`             Loads instances from an SQL query the user provided with the SqlViewerDialog, then loads the instances in a background process. |
| `void` | `setInstancesFromFile(AbstractFileLoader loader)`             Loads results from a set of instances retrieved with the supplied loader. |
| `void` | `setInstancesFromFileQ()`             Queries the user for a file to load instances from, then loads the instances in a background process. |
| `void` | `setInstancesFromURL(java.net.URL u)`             Loads instances from a URL. |
| `void` | `setInstancesFromURLQ()`             Queries the user for a URL to load instances from, then loads the instances in a background process. |
| `void` | `setLog(Logger newLog)`             Sets the Logger to receive informational messages |
| `void` | `undo()`             Reverts to the last backed up version of the dataset. |

**Methods inherited from class javax.swing.JPanel**

`getAccessibleContext, getUI, getUIClassID, setUI, updateUI`

**Methods inherited from class javax.swing.JComponent**

`addAncestorListener, addNotify, addVetoableChangeListener, computeVisibleRect, contains, createToolTip, disable, enable, firePropertyChange, firePropertyChange, firePropertyChange, getActionForKeyStroke, getActionMap, getAlignmentX, getAlignmentY, getAncestorListeners, getAutoscrolls, getBaseline, getBaselineResizeBehavior, getBorder, getBounds, getClientProperty, getComponentPopupMenu, getConditionForKeyStroke, getDebugGraphicsOptions, getDefaultLocale, getFontMetrics, getGraphics, getHeight, getInheritsPopupMenu, getInputMap, getInputMap, getInputVerifier, getInsets, getInsets, getListeners, getLocation, getMaximumSize, getMinimumSize, getNextFocusableComponent, getPopupLocation, getPreferredSize, getRegisteredKeyStrokes, getRootPane, getSize, getToolTipLocation, getToolTipText, getToolTipText, getTopLevelAncestor, getTransferHandler, getVerifyInputWhenFocusTarget, getVetoableChangeListeners, getVisibleRect, getWidth, getX, getY, grabFocus, isDoubleBuffered, isLightweightComponent, isManagingFocus, isOpaque, isOptimizedDrawingEnabled, isPaintingForPrint, isPaintingTile, isRequestFocusEnabled, isValidateRoot, paint, paintImmediately, paintImmediately, print, printAll, putClientProperty, registerKeyboardAction, registerKeyboardAction, removeAncestorListener, removeNotify, removeVetoableChangeListener, repaint, repaint, requestDefaultFocus, requestFocus, requestFocus, requestFocusInWindow, resetKeyboardActions, reshape, revalidate, scrollRectToVisible, setActionMap, setAlignmentX, setAlignmentY, setAutoscrolls, setBackground, setBorder, setComponentPopupMenu, setDebugGraphicsOptions, setDefaultLocale, setDoubleBuffered, setEnabled, setFocusTraversalKeys, setFont, setForeground, setInheritsPopupMenu, setInputMap, setInputVerifier, setMaximumSize, setMinimumSize, setNextFocusableComponent, setOpaque, setPreferredSize, setRequestFocusEnabled, setToolTipText, setTransferHandler, setVerifyInputWhenFocusTarget, setVisible, unregisterKeyboardAction, update`

**Methods inherited from class java.awt.Container**

`add, add, add, add, add, addContainerListener, addPropertyChangeListener, applyComponentOrientation, areFocusTraversalKeysSet, countComponents, deliverEvent, doLayout, findComponentAt, findComponentAt, getComponent, getComponentAt, getComponentAt, getComponentCount, getComponents, getComponentZOrder, getContainerListeners, getFocusTraversalKeys, getFocusTraversalPolicy, getLayout, getMousePosition, insets, invalidate, isAncestorOf, isFocusCycleRoot, isFocusCycleRoot, isFocusTraversalPolicyProvider, isFocusTraversalPolicySet, layout, list, list, locate, minimumSize, paintComponents, preferredSize, printComponents, remove, remove, removeAll, removeContainerListener, setComponentZOrder, setFocusCycleRoot, setFocusTraversalPolicy, setFocusTraversalPolicyProvider, setLayout, transferFocusBackward, transferFocusDownCycle, validate`

**Methods inherited from class java.awt.Component**

`action, add, addComponentListener, addFocusListener, addHierarchyBoundsListener, addHierarchyListener, addInputMethodListener, addKeyListener, addMouseListener, addMouseMotionListener, addMouseWheelListener, bounds, checkImage, checkImage, contains, createImage, createImage, createVolatileImage, createVolatileImage, dispatchEvent, enable, enableInputMethods, firePropertyChange, firePropertyChange, firePropertyChange, firePropertyChange, firePropertyChange, getBackground, getBounds, getColorModel, getComponentListeners, getComponentOrientation, getCursor, getDropTarget, getFocusCycleRootAncestor, getFocusListeners, getFocusTraversalKeysEnabled, getFont, getForeground, getGraphicsConfiguration, getHierarchyBoundsListeners, getHierarchyListeners, getIgnoreRepaint, getInputContext, getInputMethodListeners, getInputMethodRequests, getKeyListeners, getLocale, getLocation, getLocationOnScreen, getMouseListeners, getMouseMotionListeners, getMousePosition, getMouseWheelListeners, getName, getParent, getPeer, getPropertyChangeListeners, getPropertyChangeListeners, getSize, getToolkit, getTreeLock, gotFocus, handleEvent, hasFocus, hide, imageUpdate, inside, isBackgroundSet, isCursorSet, isDisplayable, isEnabled, isFocusable, isFocusOwner, isFocusTraversable, isFontSet, isForegroundSet, isLightweight, isMaximumSizeSet, isMinimumSizeSet, isPreferredSizeSet, isShowing, isValid, isVisible, keyDown, keyUp, list, list, list, location, lostFocus, mouseDown, mouseDrag, mouseEnter, mouseExit, mouseMove, mouseUp, move, nextFocus, paintAll, postEvent, prepareImage, prepareImage, remove, removeComponentListener, removeFocusListener, removeHierarchyBoundsListener, removeHierarchyListener, removeInputMethodListener, removeKeyListener, removeMouseListener, removeMouseMotionListener, removeMouseWheelListener, removePropertyChangeListener, repaint, repaint, repaint, resize, resize, setBounds, setBounds, setComponentOrientation, setCursor, setDropTarget, setFocusable, setFocusTraversalKeysEnabled, setIgnoreRepaint, setLocale, setLocation, setLocation, setName, setSize, setSize, show, show, size, toString, transferFocus, transferFocusUpCycle`

**Methods inherited from class java.lang.Object**

`equals, getClass, hashCode, notify, notifyAll, wait, wait, wait`

**Constructor Detail**

### PreprocessPanel

```
public PreprocessPanel()
```

:   Creates the instances panel with no initial instances.


**Method Detail**

### setLog

```
public void setLog(Logger newLog)
```

:   Sets the Logger to receive informational messages

    :   **Specified by:**: `setLog` in interface `Explorer.LogHandler`
    :   **Parameters:**: `newLog` - the Logger that will now get info messages

---


### setInstances

```
public void setInstances(Instances inst)
```

:   Tells the panel to use a new base set of instances.

    :   **Specified by:**: `setInstances` in interface `Explorer.ExplorerPanel`
    :   **Parameters:**: `inst` - a set of Instances

---


### getInstances

```
public Instances getInstances()
```

:   Gets the working set of instances.

    :   **Returns:**: the working instances

---


### addPropertyChangeListener

```
public void addPropertyChangeListener(java.beans.PropertyChangeListener l)
```

:   Adds a PropertyChangeListener who will be notified of value changes.

    :   **Overrides:**: `addPropertyChangeListener` in class `java.awt.Container`
    :   **Parameters:**: `l` - a value of type 'PropertyChangeListener'

---


### removePropertyChangeListener

```
public void removePropertyChangeListener(java.beans.PropertyChangeListener l)
```

:   Removes a PropertyChangeListener.

    :   **Overrides:**: `removePropertyChangeListener` in class `java.awt.Component`
    :   **Parameters:**: `l` - a value of type 'PropertyChangeListener'

---


### saveWorkingInstancesToFileQ

```
public void saveWorkingInstancesToFileQ()
```

:   Queries the user for a file to save instances as, then saves the
    instances in a background process. This is done in the IO
    thread, and an error message is popped up if the IO thread is busy.

---


### saveInstancesToFile

```
public void saveInstancesToFile(AbstractFileSaver saver,
                                Instances inst)
```

:   saves the data with the specified saver

    :   **Parameters:**: `saver` - the saver to use for storing the data: `inst` - the data to save

---


### setInstancesFromFileQ

```
public void setInstancesFromFileQ()
```

:   Queries the user for a file to load instances from, then loads the
    instances in a background process. This is done in the IO
    thread, and an error message is popped up if the IO thread is busy.

---


### setInstancesFromDBQ

```
public void setInstancesFromDBQ(java.lang.String url,
                                java.lang.String user,
                                java.lang.String pw,
                                java.lang.String query)
```

:   Loads instances from an SQL query the user provided with the
    SqlViewerDialog, then loads the instances in a background process. This is
    done in the IO thread, and an error message is popped up if the IO thread
    is busy.

    :   **Parameters:**: `url` - the database URL: `user` - the user to connect as: `pw` - the password of the user: `query` - the query for retrieving instances from

---


### setInstancesFromURLQ

```
public void setInstancesFromURLQ()
```

:   Queries the user for a URL to load instances from, then loads the
    instances in a background process. This is done in the IO
    thread, and an error message is popped up if the IO thread is busy.

---


### generateInstances

```
public void generateInstances()
```

:   sets Instances generated via DataGenerators (pops up a Dialog)

---


### setInstancesFromFile

```
public void setInstancesFromFile(AbstractFileLoader loader)
```

:   Loads results from a set of instances retrieved with the supplied loader.
    This is started in the IO thread, and a dialog is popped up
    if there's a problem.

    :   **Parameters:**: `loader` - the loader to use

---


### setInstancesFromDB

```
public void setInstancesFromDB(InstanceQuery iq)
```

:   Loads instances from a database

    :   **Parameters:**: `iq` - the InstanceQuery object to load from (this is assumed to have been already connected to a valid database).

---


### setInstancesFromURL

```
public void setInstancesFromURL(java.net.URL u)
```

:   Loads instances from a URL.

    :   **Parameters:**: `u` - the URL to load from.

---


### addUndoPoint

```
public void addUndoPoint()
                  throws java.lang.Exception
```

:   Backs up the current state of the dataset, so the changes can be undone.

    :   **Throws:**: `java.lang.Exception` - if an error occurs

---


### undo

```
public void undo()
```

:   Reverts to the last backed up version of the dataset.

---


### edit

```
public void edit()
```

:   edits the current instances object in the viewer

---


### setExplorer

```
public void setExplorer(Explorer parent)
```

:   Sets the Explorer to use as parent frame (used for sending notifications
    about changes in the data)

    :   **Specified by:**: `setExplorer` in interface `Explorer.ExplorerPanel`
    :   **Parameters:**: `parent` - the parent frame

---


### getExplorer

```
public Explorer getExplorer()
```

:   returns the parent Explorer frame

    :   **Specified by:**: `getExplorer` in interface `Explorer.ExplorerPanel`
    :   **Returns:**: the parent

---


### capabilitiesFilterChanged

```
public void capabilitiesFilterChanged(Explorer.CapabilitiesFilterChangeEvent e)
```

:   method gets called in case of a change event

    :   **Specified by:**: `capabilitiesFilterChanged` in interface `Explorer.CapabilitiesFilterChangeListener`
    :   **Parameters:**: `e` - the associated change event

---


### getTabTitle

```
public java.lang.String getTabTitle()
```

:   Returns the title for the tab in the Explorer

    :   **Specified by:**: `getTabTitle` in interface `Explorer.ExplorerPanel`
    :   **Returns:**: the title of this tab

---


### getTabTitleToolTip

```
public java.lang.String getTabTitleToolTip()
```

:   Returns the tooltip for the tab in the Explorer

    :   **Specified by:**: `getTabTitleToolTip` in interface `Explorer.ExplorerPanel`
    :   **Returns:**: the tooltip of this tab

---


### main

```
public static void main(java.lang.String[] args)
```

:   Tests out the instance-preprocessing panel from the command line.

    :   **Parameters:**: `args` - ignored


---


|  |  |  |
| --- | --- | --- |
| **Overview** \| **Package** \| **Class** \| **Tree** \| **Deprecated** \| **Index** \| **Help** \| **Weka's home** | |  |
| **PREV CLASS**   **NEXT CLASS** | **FRAMES**    **NO FRAMES**     **All Classes** |  |
| SUMMARY: NESTED \| FIELD \| CONSTR \| METHOD | DETAIL: FIELD \| CONSTR \| METHOD |  |


---
